# Intestinal Absorption of Fucoidan Extracted from the Brown Seaweed, *Cladosiphon okamuranus*

**DOI:** 10.3390/md13010048

**Published:** 2014-12-25

**Authors:** Takeaki Nagamine, Kyoumi Nakazato, Satoru Tomioka, Masahiko Iha, Katsuyuki Nakajima

**Affiliations:** 1 Graduate School of Health Sciences, Gunma University, Maebashi, Gunma 371-8514, Japan; E-Mails: nkyoumi@gunma-u.ac.jp (K.N.); besu13bikky@yahoo.co.jp (S.T.); nakajimak05@ybb.ne.jp (K.N.); 2 South Product, Co., Ltd., Uruma, Okinawa 904-1103, Japan; E-Mail: miha.south@nifty.com

**Keywords:** fucoidan, intestinal absorption, macrophage, Kupffer cells, Caco-2 cells

## Abstract

The aim of this study was to examine the absorption of fucoidan through the intestinal tract. Fucoidan (0.1, 0.5, 1.0, 1.5 and 2.0 mg/mL) was added to Transwell inserts containing Caco-2 cells. The transport of fucoidan across Caco-2 cells increased in a dose-dependent manner up to 1.0 mg/mL. It reached a maximum after 1 h and then rapidly decreased. In another experiment, rats were fed standard chow containing 2% fucoidan for one or two weeks. Immunohistochemical staining revealed that fucoidan accumulated in jejunal epithelial cells, mononuclear cells in the jejunal lamina propria and sinusoidal non-parenchymal cells in the liver. Since we previously speculated that nitrosamine may enhance the intestinal absorption of fucoidan, its absorption was estimated in rats administered *N*-butyl-*N*-(4-hydroxybutyl) nitrosamine (BBN) in their drinking water. Rats were fed 0.2% fucoidan chow (BBN + 0.2% fucoidan rats), 2% fucoidan chow (BBN + 2% fucoidan rats) and standard chow for eight weeks. The uptake of fucoidan through the intestinal tract seemed to be low, but was measurable by our ELISA method. Fucoidan-positive cells were abundant in the small intestinal mucosa of BBN + 2% fucoidan rats. Most fucoidan-positive cells also stained positive for ED1, suggesting that fucoidan was incorporated into intestinal macrophages. The uptake of fucoidan by Kupffer cells was observed in the livers of BBN + 2% fucoidan rats. In conclusion, the absorption of fucoidan through the small intestine was demonstrated both *in vivo* and *in vitro*.

## 1. Introduction

Fucoidan is a complex sulfated polysaccharide derived from marine brown seaweed. It exhibits many different biological properties, including anti-inflammatory, anti-coagulant, anti-thrombotic, anti-adhesive, anti-angiogenic, anti-viral, anti-tumor and anti-oxidant activities [1,2,3,4,5]. The biological properties of fucoidan vary depending on species, molecular weight, composition, structure and the route of administration.

Although previous studies have reported its biological effects following its oral ingestion, few have examined the uptake and fate of fucoidan [6,7,8]_._ Since high molecular weight polysaccharides are not considered to be absorbed orally, difficulties have been associated with understanding how systemic effects occur. Michel *et al.* [9] suggested that fucoidan was not fermented by human bacterial flora and was wholly excreted after its oral administration. The properties of fucoidan have been assumed to reflect the various biological reactions induced in the gastrointestinal tract after its administration [10,11]. On the other hand, Irhimeh *et al.* [6] measured the serum fucoidan levels by the competitive ELISA and showed that the absorption rate of fucoidan at the intestine was 0.6%, which was much higher than observed by us [8]. The difference in the serum fucoidan levels may be accounted for by difference in the source of fucoidan and the antibody used. Nakazato *et al.* [12] reported that nitrosamine may enhance the intestinal absorption of fucoidan in animal models, but the mechanism for increased uptake has not been clarified yet. We recently raised a specific antibody for fucoidan extracted from *Cladosiphon okamuranus* (Okinawa Mozuku), a traditional food in Japan, and established a sandwich ELISA method for a fucoidan assay [8]. Using this ELISA method, we assayed fucoidan concentrations in human serum after the oral administration of fucoidan and examined them in seven out of 10 men approximately 6 h after dosing. The concentration of fucoidan was higher in the urine than in the serum. However, the uptake and fate of fucoidan across the intestinal tract has not yet been elucidated in detail.

In this study, we investigated whether fucoidan was absorbable in an *in vivo* rat experiment, together with an *in vitro* experiment on its absorption characteristics in Caco-2 cells.

## 2. Results

### 2.1. Transport of Fucoidan across Caco-2 Cells

**Figure 1:**
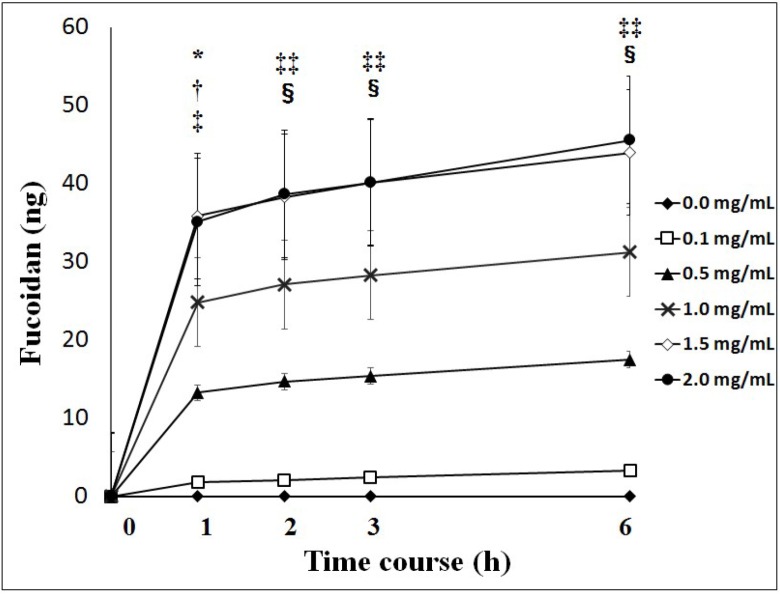
Cumulative values of fucoidan across Caco-2 cells. Cumulative values of fucoidan were significantly higher with 1.0, 1.5 and 2.0 mg/mL of fucoidan than with 0.1 and 0.5 mg/mL of fucoidan. Results show the mean ± S.E of 5 experiments. * *p <* 0.05 0.1 mg/mL of fucoidan *vs.* 1.0 mg/mL of fucoidan; **^†^** *p <* 0.01 0.1 mg/mL of fucoidan *vs.* 1.5 and 2.0 mg/mL of fucoidan; **^‡^** *p <* 0.05 0.5 mg/mL of fucoidan *vs.* 1.5 and 2.0 mg/mL of fucoidan; **^‡‡^** *p <* 0.01 0.1 mg/mL of fucoidan *vs.* 1.0, 1.5 and 2.0 mg/mL of fucoidan. **^§^** *p <* 0.01 0.5 mg/mL of fucoidan *vs.* 1.5 and 2.0 mg/mL of fucoidan. Statistical analyses were performed using two-way ANOVA.

Fucoidan was transported across Caco-2 cells during the experiment at different apical loadings. The transport of fucoidan across Caco-2 cells reached a maximum after 1 h and then decreased to basal levels after 3 h. The cumulative transport of fucoidan to the basolateral chamber during the experiment was significantly higher with 1.0, 1.5 and 2.0 mg/mL of fucoidan than with 0.1 and 0.5 mg/mL of fucoidan after the treatment (Figure 1).

The apparent permeability coefficient (*P*app) was very low, ranging from 0.16 ± 0.06 to 0.24 ± 0.03 (×10^−^^7^ cm/s) (Table 1). 

**Table 1:** Apparent permeability for the transport of fucoidan across Caco-2 cells.

Dose of Fucoidan (mg/mL)	Apparent Permeability Coefficient *P*_app_ (×10^−7^ cm/s)
0.1	0.19 ± 0.06
0.5	0.24 ± 0.03
1.0	0.22 ± 0.02
1.5	0.22 ± 0.06
2.0	0.16 ± 0.02

Data represent the mean ± S.E. of five experiments; No significant differences were observed among the five groups; Statistical analyses were performed using one-way ANOVA.

The percentage of fucoidan transported across Caco-2 cells for 1 h after the treatment was similar among the five groups (Table 2).

**Table 2:** Percentage of fucoidan transported across Caco-2 cells.

Dose of Fucoidan (mg/mL)	% of Fucoifan Transported for 1 h after the Treatment
0.1	0.0076 ± 0.0003
0.5	0.0094 ± 0.0008
1.0	0.0087 ± 0.0010
1.5	0.0082 ± 0.0051
2.0	0.0062 ± 0.0025

Data represent the mean ± S.E. of five experiments; No significant differences were observed among the five groups; Statistical analyses were performed using one-way ANOVA.

### 2.2. Fucoidan Levels in the Serum and Liver from Rats Fed 2% Fucoidan Chow

Fucoidan levels were assayed in the serum and liver of rats fed 2% fucoidan chow (Table 3). Serum fucoidan was detected in rats fed 2% fucoidan chow for one week (one-week fucoidan rat) and those fed 2% fucoidan chow for two weeks (two-week fucoidan rat). Fucoidan was not determined in serum from rats fed standard chow for one week (control rat). Fucoidan was detected in the livers of both the one-week fucoidan rat and two-week fucoidan rat, but not in the control rat.

**Table 3:** Fucoidan levels in ratas fed 2% fucoidan chow or standard chow.

	Serum (ng/mL)	Liver (ng/mg Protein)
Control rat (*n* = 2)		
No.1	<1.0 *	<1.0 *
No.2	<1.0 *	<1.0 *
One week-fucoidan rat (*n* = 2)		
No.1	2.7	17.9
No.2	1.7	13.4
Two weeks-fucoidan rat (*n* = 2)		
No.1	3.0	20.1
No.2	2.4	12.8

Control rat fed standard chow without fucoidan for one week; One week-fucoidan rat fed 2% fucoidan chow for one week; Two weeks-fucoidan rat fed 2% fucoidan chow for two weeks; <1.0 *: Below the detection limit of the fucoidan ELISA assay.

### 2.3. Immunohistochemistry for Fucoidan in the Small Intestine

**Figure 2:**
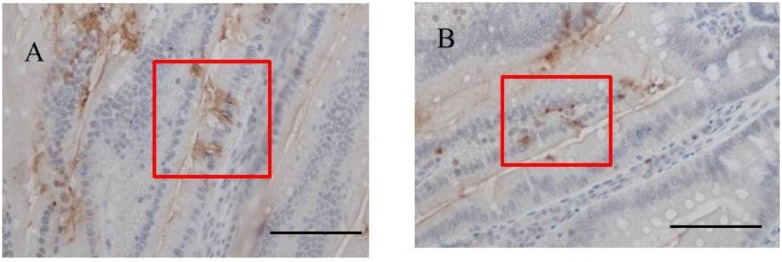

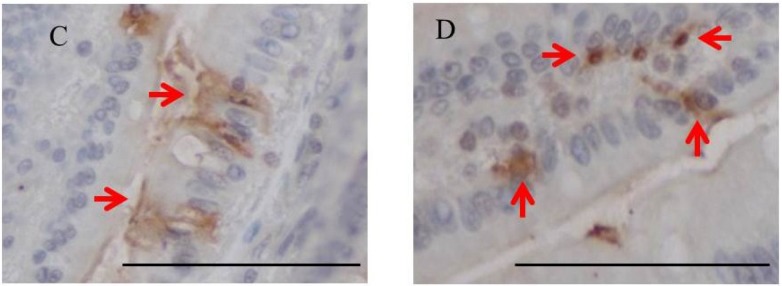
Immunohistochemistry for fucoidan in the jejunum from one-week fucoidan rat (*n =* 2). (**A**) Fucoidan stained diffusely in the epithelial cells of the jejunum (×400). Enlargement of the red frame (**C**). (**B**) Some mononuclear cells stained positively for fucoidan in the lamina propria of the jejunum (×400). Enlargement of the red frame (**D**). Arrows indicate positive staining for fucoidan. Bars: 50 μm.

Immunohistochemistry for fucoidan showed that it was incorporated into some epithelial cells in the jejunum from rats fed 2% fucoidan for one week (one-week fucoidan rat), in which epithelial cells stained diffusely for fucoidan (Figure 2). Linear staining was also observed in the gap between adjacent epithelial cells. Fucoidan was detected in round mononuclear cells in the lamia propria of the jejunum; however, staining for fucoidan was very weak in the ileum.

**Figure 3:**
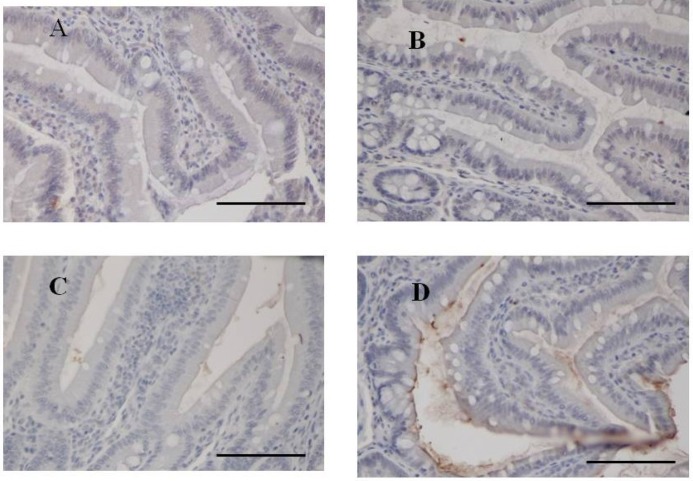
Immunohistochemistry for fucoidan in the small intestine from two-week fucoidan rat (*n =* 2) and control rat (*n =* 2). Fucoidan was faintly stained in the epithelial cells of ileum from two-week fucoidan rat. It was not stained in the jejunum and ileum from control rat. (**A**) A jejunum specimen from a control rat (×400); (**B**) An ileum specimen from a control rat (×400); (**C**) A jejunum specimen from a two-week fucoidan rat (×400); (**D**) An ileum specimen from a two-week fucoidan rat (×400). Bars: 50 μm.

It was also faintly detected in the ileum from rats fed 2% fucoidan for two weeks (two-week fucoidan rat) and was absent in those from rats fed standard chow (control rat) (Figure 3).

### 2.4. H & E Staining and Immunohistochemistry for Fucoidan in the Liver of Rats Fed Fucoidan Chow or Standard Chow

**Figure 4:**
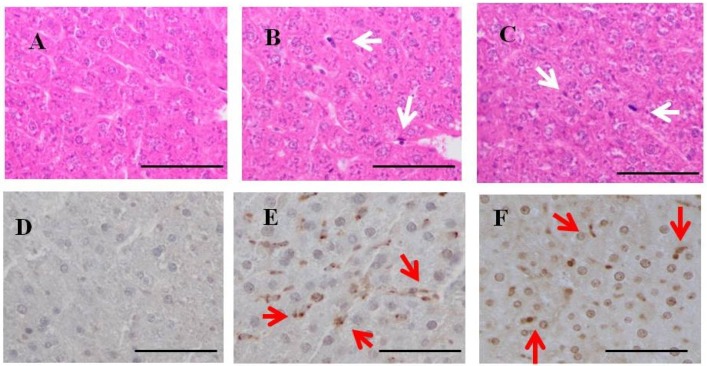
H & E staining and immunohistochemistry for fucoidan in the liver of rats fed fucoidan chow or standard chow. H & E staining: (**top**). (**A**) A liver specimen from a control rat (×400); (**B**) A liver specimen from a one-week fucoidan rat (×400); (**C**) A liver specimen from a two-week fucoidan rat (×400). White arrows indicate Kupffer cells. Immunohistochemistry for fucoidan: (**bottom**) Fucoidan was apparent in sinusoidal non-parenchymal cells in the liver from one week-week fucoidan rat and two-week fucoidan rat, but not in control rats; (**D**) A liver specimen from a control rat (×400); (**E**) A liver specimen from a one-week fucoidan rat (×400); (**F**) A liver specimen from a two-week fucoidan rat (×400). Red arrows indicate positive staining for fucoidan. Bars: 50 μm.

Hepatic H & E staining (Figure 4) showed that Kupffer cells were mildly mobilized in the sinusoids of hepatic lobules in one-week fucoidan rat (*n =* 2) and two-week fucoidan rat (*n =* 2), but not in control rat (*n =* 2) (upper panels).

Immunostaining for fucoidan in the livers (Figure 4) was elliptical or clubbed in non-parenchymal cells in sinusoids, but not in parenchymal cells. Fucoidan was apparent in the liver from one-week fucoidan rat and two-week fucoidan rat, but not stained in the livers of control rat (lower panels).

### 2.5. Fucoidan Levels in BBN-Ingesting Rats Fed Fucoidan Chow

Fucoidan levels in serum and livers were assayed in BBN-ingesting rats fed fucoidan chow. Serum fucoidan levels were slightly elevated in BBN + 0.2% fucoidan rats and BBN + 2% fucoidan rats. No significant differences were observed among the three groups. Hepatic fucoidan levels were high in the order of BBN rats, BBN + 0.2% fucoidan rats and BBN + 2% fucoidan rats. A significant difference was observed between BBN + 2% fucoidan rats and BBN rats (Table 4).

**Table 4:** Fucoidan levels in BBN-ingested rats fed fucoidan chow.

	Serum (ng/mL)	Liver (ng/mg Protein)
BBN rats (*n* = 4)	4.7 ± 1.4	0.7 ± 0.4
BBN + 0.2% fucoidan rats (*n* = 4)	8.4 ± 2.9	36.9 ± 11.5
BBN + 2% fucoidan rats (*n* = 4)	8.6 ± 2.9	67.9 ± 14.5 *

BBN rats fed standard chow for 8 weeks; BBN + fucoidan rats fed standard chow containing fucoidan for 8 weeks; Data represent the mean ± S.E. of 4 rats; * *p* < 0.05 significantly different from BBN rats; Statistical analyses were performed using one-way ANOVA.

### 2.6. Immunohistochemistry for Fucoidan in the Small Intestines of BBN-Ingesting Rats Fed Fucoidan Chow

**Figure 5:**
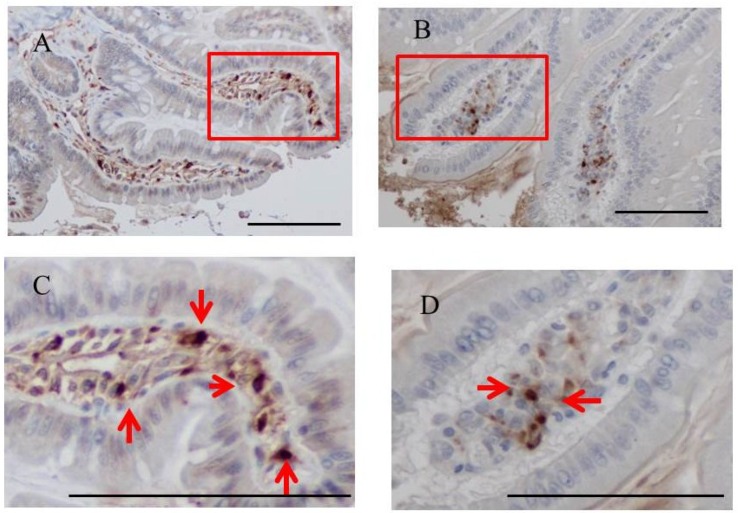
Immunohistochemistry for fucoidan in the small intestines of BBN + 2% fucoidan rats (*n =* 4). (**A**) A small number of fucoidan-positive cells was detected in the lamina propria of the jejunum (×400). Enlargement of the red frame (**C**). (**B**) Fucoidan-positive cells were abundant in mononuclear cells in the lamina propria of the ileum from BBN + 2% fucoidan rats (×400). Enlargement of the red frame (**D**) Arrows indicate fucoidan-positive cells. Bars: 50 μm.

Fucoidan was stained immunohistochemically in the small intestines of BBN-ingesting rats fed fucoidan chow. Fucoidan-positive cells were abundant in the ileum of BBN + 2% fucoidan rats. The cells that were positive for fucoidan were mononuclear, round in shape and were mainly scattered in the lamina propria of the ileum. A small number was detected in the mucosa of the jejunum (Figure 5).

Staining for fucoidan was weak in tissue samples from BBN + 0.2% fucoidan rats and was not observed in tissue samples from BBN rats.

### 2.7. Double Staining for Fucoidan and ED1 in the Ileum of BBN-Ingesting Rats

**Figure 6:**
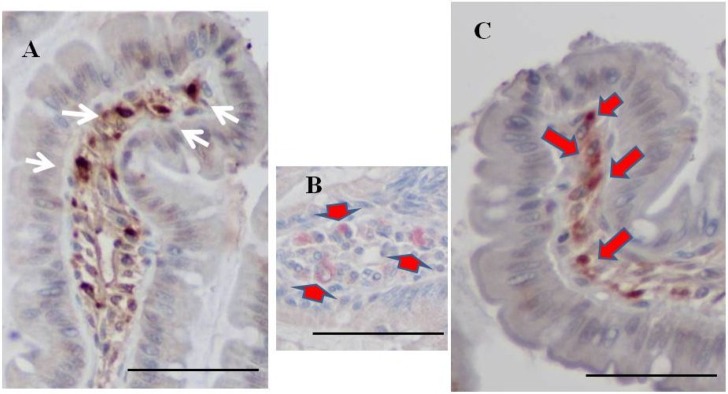
Double staining for fucoidan and ED1. (**A**) A representative case of single staining for fucoidan. Fucoidan stained dark brown in mononuclear cells in the lamina propria of the ileum from a BBN + 2% fucoidan rat (white arrows); (**B**) A representative case of single staining for ED1. ED1 stained pink in round mononuclear cells in the lamina propria of the ileum from a BBN rat (arrows); (**C**) Some mononuclear cells in the lamina propria of the ileum from BBN + 2% fucoidan rats were positive for both fucoidan and ED1 staining. Cells were stained pink by the ED1 stain and brown by fucoidan staining (red arrows). Bars: 50 μm.

We performed double staining for fucoidan and the macrophage marker, ED1, using a whole-mount in order to determine whether mononuclear cells staining for fucoidan were macrophages. ED1-positive cells were observed occasionally in BBN rats. Fucoidan and ED1 double-positive cells were scattered in the lamina propria of the ileum from BBN + 2% fucoidan rats (Figure 6).

**Figure 7:**
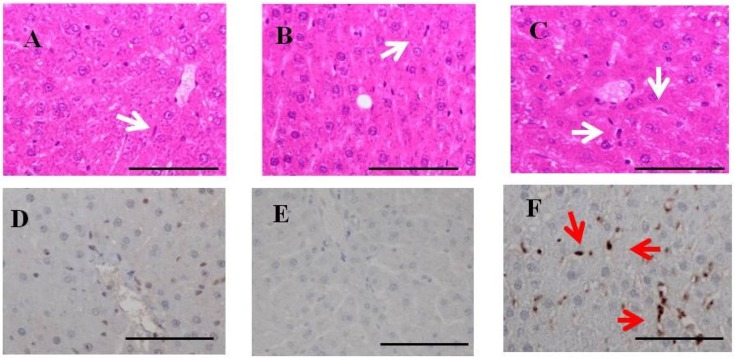
H & E staining and immunohistochemistry for fucoidan in the liver of BBN-ingesting rats fed fucoidan chow or standard chow. H & E staining: (**top**). (**A**) A liver specimen from a BBN rat (×400); (**B**) A liver specimen from a BBN + 0.2% fucoidan rat (×400); (**C**) A liver specimen from a BBN + 2%fucoidan rat (×400); white arrows indicate Kupffer cells. Immunohistochemistry for fucoidan: (**bottom**); (**D**) A liver specimen from a BBN rat (×400); (**E**) A liver specimen from a BBN + 0.2% fucoidan rat (×400); (**F**) A liver specimen from a BBN + 2% fucoidan rats (×400). Red arrows indicate positive staining for fucoidan. Bars: 50 μm.

### 2.8. H & E Staining and Immunohistochemistry for Fucoidan in the Livers of BBN-Ingesting Rats

Hepatic H & E staining (Figure 7) demonstrated that Kupffer cells were (clearly) mobilized in BBN + 2% fucoidan (*n =* 4) and less frequently in BBN + 0.2% fucoidan rats (*n =* 4) and BBN rats (*n =* 4). Swollen Kupffer cells were scattered in the hepatic lobules of livers from BBN + 2% fucoidan rats. No obvious liver damage was observed in BBN-ingesting rats with or without fucoidan (upper panels).

Immunohistochemistry for fucoidan in the livers of BBN-ingesting rats fed fucoidan chow (Figure 7) is shown in the lower panels. Fucoidan-positive cells were abundant in sinusoidal non-parenchyma cells, but were absent in parenchymal cells of the livers from BBN + 2% fucoidan rats. Fucoidan was not stained in the livers of BBN + 0.2% fucoidan rats and BBN rats.

### 2.9. Double Staining for Fucoidan and ED1 on the Liver of BBN-Ingesting Rats

**Figure 8:**
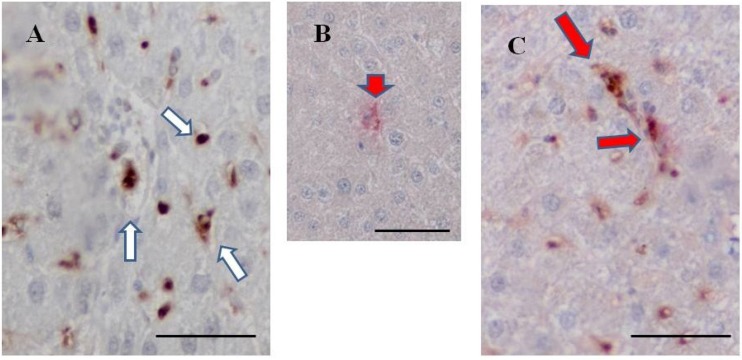
Double staining for fucoidan and ED1 in the livers of BBN + 2% fucoidan rats. (**A**) A representative case of single staining for fucoidan. Fucoidan stained dark brown in Kupffer cells in the liver of a BBN + 2% fucoidan rat (white arrows); (**B**) A representative case of single staining for ED1. ED1 stained pink in Kupffer cells in the liver of a BBN rat (head arrows); (**C**) A representative case of double staining for fucoidan and ED1. Some Kupffer cells in the sinusoids of hepatic lobules from BBN + 2% fucoidan rats were positive for both fucoidan and ED1 staining (red arrows). Bars: 50 μm.

Double immunostaining for fucoidan and ED1 (a marker for Kupffer cells) was also performed in the livers of BBN + 2% fucoidan rats. Double-positive cells for fucoidan and ED1 were clearly detected in the sinusoids of hepatic lobules, suggesting the uptake of fucoidan by Kupffer cells (Figure 8).

## 3. Discussion

The present study demonstrated that high molecular weight fucoidan, extracted from *Cladosiphon okamuranus* (Okinawa Mozuku), was absorbed through intestinal epithelial cells, both *in vivo* and *in vitro*. The Papp value of fucoidan was smaller than that of other polysaccharides, such as low molecular weight heparin (average MW: 4.125 kDa) [13]. The permeabilities of the sulfated polysaccharides of heparin and hyaluronan have been shown to inversely increase with molecular size and were also dose dependent [13,14]. Since the fucoidan used in this study consisted of a heterogeneous molecular weight (main MW: 28.8 kDa) [14], its transport across Caco-2 cells should be restrictive and disturbed. As expected, the percentage of fucoidan transported across Caco-2 cells was very low for 1 h after the treatment. Kimura *et al.* [15] more recently confirmed our results in which the percentage of fucoidan transported across Caco-2 cells was 0.001 ± 0.001 for 2 h after the treatment.

Fucoidan can pass the intestinal barrier through two parallel routes; transcellular and paracellular. The transport of fucoidan across Caco-2 cells reached a maximum after 1 h and then rapidly decreased after the treatment. Since the saturability of transportation does not occur in simple diffusion, fucoidan may be absorbed via a transporter or pinocytosis [16,17]. However, the permeation of high molecular weight polysaccharides, such as fucoidan, through the paracellular pathway was previously shown to be restricted by tight junctions [18]. Future studies to elucidate the absorption mechanism across Caco-2 cells are warranted; therefore, the receptor-dependent absorption of fucoidan needs to be examined in more detail.

The small intestine and liver stained positively for fucoidan after its oral administration to rats. We observed the incorporation of fucoidan in the epithelial cells of the jejunum from rats fed 2% fucoidan chow for one week, suggesting that fucoidan may be absorbed through the primary intestinal cells. In addition, round mononuclear cells residing in the lamina propria of the jejunum stained positively for fucoidan. In contrast, staining for fucoidan was weak in ileum specimens. In spite of the detection of fucoidan in the liver, decisive fucoidan-positive epithelial cells were not found in intestinal specimens removed from the two-week fucoidan rats. In this study, we only examined one specimen each of the jejunum and ileum, 1 cm in length, from every rat; therefore, we need to examine the whole small intestine in order to determine where fucoidan is fundamentally absorbed. Since the experiment using fucoidan-ingesting rats without BBN was conducted as a preliminary test for the BBN + fucoidan tests, We only examined four rats fed 2% fucoidan chow in this study; hence, differences in the absorption of fucoidan through the jejunum and ileum need to be clarified using a sufficient number of rats.

We previously showed that fucoidan staining was more apparent in the livers of diethylnitrosamine-treated rats than in control rats after its oral administration [12]. This finding suggested that nitrosamine, a chemical carcinogen, may be able to facilitate the intestinal permeability of fucoidan. In order to elucidate the effects of orally administered nitrosamine on the absorption of fucoidan, we performed experiments using BBN-ingesting rats. We found that serum fucoidan levels were higher in BBN-ingesting rats fed 2% fucoidan than in rats fed 2% fucoidan, suggesting that BBN facilitates the intestinal absorption of fucoidan. As described above, fucoidan may be absorbed through a transporter or via pinocytosis; therefore, the relevance of BBN to the absorption of fucoidan via transcellular or paracellular routes should be clarified in future studies.

Fucoidan levels in the liver were significantly higher in BBN + 2% fucoidan rats than in BBN rats and were slightly elevated in the serum of BBN + 2% fucoidan rats (Table 4). Fucoidan was unexpectedly detected in BBN-ingesting rats fed standard chow. Our polyclonal anti-fucoidan antibody recognized fucoidan from *C. okamuranus* [8]*.* According to its nature of being a polyclonal antibody, this antibody may cross-react with some kind of antigen besides fucoidan. As shown in Table 3 and Table 4, fucoidan was not detected in serum of rats fed standard chow, but was detected in serum of BBN-ingesting rats fed standard chow. The findings suggest the presence of an unknown product that may cross-react with anti-fucoidan antibody after BBN administration.

Fucoidan-positive cells were observed in the intestinal mucosa of BBN + 2% fucoidan rats and were particularly prominent in ileum specimens. We characterized the fucoidan-positive cells observed in the intestinal mucosa. Macrophages are one of the most abundant leukocytes in the intestines of all mammalian species. Since fucoidan is a ligand for macrophage scavenger receptor-A, this polysaccharide may bind and be incorporated into intestinal macrophages [19]. To verify this hypothesis, we conducted double immunostaining for fucoidan and ED1, a macrophage marker. The results obtained showed that some fucoidan-positive cells were also positive for ED1, which suggested the internalization of fucoidan in macrophages. Intestinal macrophages are essential for local homeostasis and maintaining a balance between commensal microbiota and the host. Macrophages also play essential roles in inflammation and protective immunity [20,21,22]. Matsumoto *et al*. [23] reported that oral administration of fucoidan improves murine chronic colitis by downregulating the synthesis of IL-6 and IFN-γ in the colonic epithelial cells and lamina propria. In the present study, fucoidan-laden macrophages in intestinal mucosa were more visible in BBN-ingesting rats than BBN-non ingested rats. These findings suggest that fucoidan may attenuate intestinal inflammation promoted by macrophages. Further studies are required to verify whether intestinal macrophages change from peaceful regulators to powerful aggressors when they bind to fucoidan.

Strong staining for fucoidan was observed in sinusoidal non-parenchymal cells in the livers of rats fed 2% fucoidan chow. Consistent with the localization of fucoidan-positive cells, Kupffer cells were abundant in the sinusoids of hepatic lobules. In addition, double-positive staining for fucoidan and ED1 (a Kupffer cell marker) was observed in the sinusoidal non-parenchymal cells of BBN + 2% fucoidan rats. These results indicated the uptake of fucoidan by Kupffer cells. Several studies previously examined the internalization of sulfated polysaccharides in Kupffer cells [24,25,26]. β-glucan, injected intra-peritoneally, was mainly distributed in the liver and spleen, and Kupffer cells have been shown to play an important role in the metabolic degradation of β-glucans [24]. The uptake of heparin in isolated rat Kupffer cells was mediated by scavenger receptors, and the internalization of heparin, as well as surface binding to Kupffer cells was inhibited by a scavenger receptor ligand of fucoidan [25,26]. Several studies implied the inhibitory effects of fucoidan on scavenger receptor A in Kupffer cells [27,28,29], which suggests that scavenger receptors may be responsible for the uptake of fucoidan by Kupffer cells.

As shown in the present study, evaluating the tissue distribution of fucoidan after its oral administration indicated that it preferentially accumulated in the liver, accompanied by low systemic blood levels. A similar metabolic pattern was also reported for orally administered heparin, in which high-molecular-weight heparin was rapidly eliminated from the plasma according to first-order kinetics, whereas low-molecular-weight heparin was mostly excreted in the urine because of it small first-order kinetics [30,31]. The sulfated polysaccharides of heparin and fucoidan have a lot in common with regards to their heterogeneous molecular weight and anionic characteristics [32,33]. Given the similar characteristics of fucoidan and heparin, the pharmacokinetics of fucoidan may resemble those of heparin. If so, fucoidan could be rapidly eliminated from the serum following its oral administration according to first-order kinetics, leading to low serum fucoidan levels. In addition, we previously reported that serum levels of fucoidan were one-tenth its urinary levels in humans following its oral administration, the molecular weight of fucoidan was unchanged in serum, and lower molecular weight fucoidan was excreted in urine [8]. Taken together with the present and previous findings, the uptake of large-molecular-weight fucoidan by Kupffer cells in the liver following its ingestion was rapid and resulted in a small increase in fucoidan levels in the serum. On the other hand, small molecular weight fucoidan was slowly excreted in the urine. Further studies on the pharmacokinetics of fucoidan are necessary to verify this hypothesis.

## 4. Materials and Methods

### 4.1. Chemicals and Reagents

Fucoidan extracted from *Cladosiphon okamuranus* (Lot Number A1205081) was purchased from South Product Co. (Uruma, Japan). The antibody against fucoidan was originally raised in our laboratory and used for this study [8].

*N*-butyl-*N*-(4-hydroxybutyl)nitrosamine (BBN) was purchased from Tokyo Kasei Co. (Tokyo, Japan). Histofine Simple Stain was purchased from Nichirei (Tokyo, Japan), and anti-rabbit IgG-peroxidase was from Sigma-Aldrich (St. Louis, MO, USA). Fetal bovine serum was purchased from DS Pharma Biomedical (SantaAna, CA, USA). Hank’s balanced salt solution (HBSS) was purchased from Sigma-Aldrich (St. Louis, MO, USA). ED1, an antibody for rat macrophages and Kupffer cells, was purchased from AbD SEROTEC (Oxford, U.K.). Transwell™ (12 mm in diameter with a 0.4-μm pore size, polyester membrane) was purchased from Corning Coster Corp. (Corning, NY, USA).

Standard rat chow (type MF) and fucoidan-containing MF chows (0.2% or 2% w/w) were tableted and provided by Oriental Yeast Co., Ltd. (Tokyo, Japan).

### 4.2. Transport of Fucoidan across Caco-2 Cells

The human colorectal adenocarcinoma Caco-2 cell line (EC86010202) was obtained from Dainippon Sumitomo Pharma. Co. (Osaka, Japan). The Caco-2 cells used in this study were between Passages 50 and 55. They were maintained in DMEM containing 20% FBS, 100 U/mL of penicillin, 100 μg/mL of streptomycin and 2 mmol/L of l-glutamine under 5% CO_2_ at 37 °C. Cells were seeded at a density of 4.5 × 10^5^ cells per well in Transwell inserts and cultivated with the tissue culture medium being replaced three times a week for 14 days. The integrity of the Caco-2 monolayer was determined by measuring the transepithelial electrical resistance (TEER) of the cell monolayer grown on filter support using the Millicell-ERS electrical resistance measuring system (Millipore, Bedford, MA, USA). Monolayers with TEER over 500 Ω/cm^2^ were used for the experiment.

Fucoidan for experimental use was dissolved with HBSS as 0-, 0.1-, 0.5-, 1.0-, 1.5- and 2.0-mg/mL fucoidan solutions. Before starting the fucoidan transport experiments, the medium was removed, and culture wells were washed twice with modified HBSS. A total of 0.3 mL of each fucoidan solution was then applied to each well in Transwell inserts. A total of 1.2 mL of HBSS was added to the basolateral chamber. All basolateral HBSS was collected and replaced by an equal volume of HBSS 0, 1, 2, 3 and 6 h after the fucoidan treatment. Samples were stored at −80 °C until the fucoidan assay.

*P*app (apparent permeability) was calculated using the following equation [13]:

Papp =Q/Act

where *Q* is the total amount permeated within the incubation time (μg), *A* is the membrane surface area (cm^2^), *c* is the initial concentration of fucoidan within the apical compartment (μg/cm^3^) and *t* is the total time of the experiment.

### 4.3. Fucoidan Absorption in Rats after Its Oral Administration

Six Wistar rats (male, weight 180 g) were supplied from Charles River Japan (Kanagawa, Japan). Rats were caged in the animal facility of Gunma University, with a 12-h light/dark regime. Rats were fed on standard chow containing 2% fucoidan (2% fucoidan chow) for one week (*n =* 2) or two weeks (*n =* 2), and two rats fed on standard chow served as a control. All rats had free access to tap water during the experiment.

Rats were anesthetized with ether, and blood was drawn from the inferior vena cava. The liver was surgically removed, and a random piece of each organ was fixed in Carnoy fluid. One specimen each of the jejunum and ileum, 1 cm in length from every rat, was fixed in Carnoy fluid. These specimens were then embedded in paraffin and cut in 3-μm sections for fucoidan immunostaining. Another liver specimen was fixed in 10% formalin and then embedded in paraffin for hematoxylin and eosin (H & E) staining. The remaining samples were kept frozen at −80 °C until biochemical analysis.

### 4.4. Fucoidan Absorption after Its Oral Administration to Rats That Ingested N-Butyl-N-(4-hydroxybutyl)nitrosamine (BBN)

We previously reported that fucoidan staining in the liver was more apparent in nitrosamine-treated rats than in control rats [14]. In order to accelerate the absorption of fucoidan, twelve rats had access to tap water containing 0.5% BBN, a type of nitrosamine, *ad libitum* during the experiment. Rats were randomized into 3 experimental groups of 4 rats each. Group 1, which served as BBN rats, was fed standard chow. Group 2 (BBN + 0.2% fucoidan rats) and Group 3 (BBN + 2% fucoidan rats) were fed standard chow containing 0.2% fucoidan or 2% fucoidan, respectively.

After 8 weeks of the oral ingestion of BBN together with or without fucoidan, rats were anesthetized with ether, and blood was drawn from the inferior vena cava. The liver, jejunum and ileum were surgically removed. A random piece of each organ was fixed in Carnoy fluid for fucoidan staining. Another liver specimen was fixed in 10% formalin and then embedded in paraffin for H & E staining. The remaining samples were kept frozen at −80 °C until biochemical analysis.

All of the protocols were carried out in accordance with Gunma University’s ethical guidelines for laboratory animals (Approval Number 12-058, corresponding date: 1 October 2012).

### 4.5. Fucoidan Assay

One gram of liver tissue was added to 1 mL of saline and was then homogenized using an ultrasonicator. Fucoidan concentrations in serum and tissue homogenates were measured using a sandwich ELISA method described previously [8]. The minimum detectable sensitivity of our fucoidan ELISA assay was considered to be 1 ng/mL of fucoidan.

#### 4.5.1. Fucoidan Staining

Fucoidan was stained in tissue samples using an immunohistochemical method described previously [12].

#### 4.5.2. Double Staining for Fucoidan and ED1

Double staining for fucoidan and ED1 was performed in the liver and ileum specimens from BBN + 2% fucoidan rats. After deparaffinization, sections were immersed in hot 0.01 M citrate buffer (pH 6.0), heated in a microwave oven for 5 min for antigen retrieval and were then washed 3 times with PBS. Sections were incubated with polyclonal antibodies for ED1 (1:50) for 60 min at 37 °C and were washed 3 times with PBS containing 0.05% Tween 20. They were then incubated with the secondary antibody (Histofine Simple Stain AP (Nichirei Bioscience Inc, Tokyo, Japan)) for 30 min at room temperature and washed 3 times with PBS containing 0.05% Tween 20. Section were incubated in the new fuchsine solution for 10 to 20 min at room temperature and washed with tap water for 5 min. Sections were immersed in hot 0.01 M citrate buffer (pH 6.0), heated in a microwave oven for 5 min for antigen retrieval and then washed 3 times with PBS. Sections were treated with methanol containing 3% H_2_O_2_ for 10 min at room temperature to eliminate endogenous peroxidase activity and were washed 3 times with PBS. They were incubated with polyclonal antibodies for fucoidan (1:100) for 90 min at room temperature and washed 3 times with PBS containing 0.05% Tween 20. Sections were then incubated with the secondary antibody (Histofine Simple Stain MAX-PO, Nichirei Bioscience Inc, Tokyo, Japan) for 30 min at room temperature and washed 3 times with PBS containing 0.05% Tween 20. Sections were incubated in the color reaction solution (150 mL of 0.05 M Tris-HCl buffer, 30 mg DAB, 10 μL H_2_O_2_) for 10 to 20 min at room temperature and were washed with tap water for 5 min. Sections were then counter-stained with Mayer’s hematoxylin.

### 4.6. Liver Pathology

Liver samples were cut into 3-μm sections. After deparaffinization, liver specimens were stained with H & E. Histological findings were examined under light microscopy.

### 4.7. Statistical Analysis

Data were analyzed by a two-way analysis of variance (ANOVA) or a one-way ANOVA using SPSS (SPSS Inc., Chicago, IL, USA), followed by Tukey’s test for multiple comparisons. Probabilities of *p <* 0.05 were regarded as significant.

## 5. Conclusions

In the present study, we demonstrated that high molecular weight fucoidan, extracted from *Cladosiphon okamuranus* (Okinawa Mozuku), was absorbed through small intestine, both *in vivo* and *in vitro*. This knowledge will enable us to comprehensively understand the biological effects of fucoidan following its oral ingestion.
